# Long Term Results of a Rotating Hinge Total Knee Prosthesis With Carbon-Fiber Reinforced Poly-Ether-Ether-Ketone (CFR-PEEK) as Bearing Material

**DOI:** 10.3389/fbioe.2022.845859

**Published:** 2022-03-04

**Authors:** Klemens Vertesich, Kevin Staats, Christoph Böhler, Richard Koza, Richard Lass, Alexander Giurea

**Affiliations:** Department of Orthopedics and Trauma Surgery, Medical University of Vienna, Vienna, Austria

**Keywords:** rotating hinge, carbon-fiber reinforced poly-ether-ether-ketone (CFR-PEEK), TKA, revision TKA, knee joint instability, severe osteoarthritis, complex revision surgery

## Abstract

**Background:** The use of rotating hinge (RH) prostheses for severe primary as well as revision arthroplasty is widely established. Due to the steadily increasing number of RH prostheses, we aimed to assess the complication frequencies, complication types and clinical outcomes of a modern RH hinge prosthesis using a new bearing material with a minimum follow-up of 7 years.

**Methods:** Fifty-six consecutive patients who received the EnduRo^®^ RH prosthesis using carbon-fiber reinforced poly-ether-ether-ketone (CFR-PEEK) as bearing material were included in this prospective study: 21 patients (37.5%) received the prosthesis as a primary total knee arthroplasty (TKA) and 35 patients (62.5%) underwent revision total knee arthroplasties (rTKA). Clinical and radiographic examinations were performed preoperatively, postoperatively after three and 12 months and annually thereafter. Clinical scores were documented for each patient. Competing risk analysis was assessed with respect to indication and failure mode.

**Results:** Knee Society Score (KSS), Western Ontario and McMaster Osteoarthritis Index (WOMAC), Oxford Knee Score (OKS) and range of motion (ROM) improved significantly compared to preoperative values (*p* < 0.0001). The overall cumulative incidence for revision surgery was 23.6% at 7 years and the cumulative incidence for complications associated with failure of the prothesis was 5.6% at 7 years, respectively. Complications occurred more frequently in the revision group (*p* = 0.002).

**Conclusion:** The evaluated RH prosthesis provided reliable and durable results for a minimum follow-up of 7 years. Prosthesis survival was successful considering the complexity of the cases. The use of this RH system in primary patients showed high survival rates and long-term functional outcomes and clinical outcomes proved to be satisfying in both revision and primary cases. No adverse events were associated with the new bearing material CFR-PEEK.

## Introduction

Constantly rising numbers of revision total knee arthroplasties (rTKA) represent a main clinical and economic burden for orthopedic surgery (Kurtz et al., 2007; Bhandari et al., 2012; Delanois et al., 2017). Reasonable and functional reconstruction, especially in complex rTKA, requires a higher degree of constraint (Kearns et al., 2018). For cases with substantial bone loss and ligamentous instability, hinged knee prostheses are a viable option for the reconstructing the joint and the restoring of its functionality (Callaghan et al., 2005). In complex primary total knee arthroplasty (TKA) with severe deformities, such as excessive valgus/varus malalignment, post-traumatic defects or joint deformity due to neuromuscular diseases and rheumatoid arthritis, hinged knee prostheses may represent a reliable choice for joint reconstruction (Gehrke et al., 2014).

Initially introduced designs of hinged knee prostheses restricted motion to only one plane. This caused high shear stress on bone-implant interfaces and led to disappointing and unacceptably high failure rates (Freeman, 1973; Jones et al., 1979; Barrack, 2001). Consequently, fixed hinged knee prostheses developed into an unfavorable option for reconstruction. Subsequent improvements of hinged prostheses introduced a rotational axis allowing up to 20 degrees of internal and external rotation of the hinge (Kester et al., 1988; Barrack, 2002). Additionally, improvements of the trochlear groove decreasing patellar maltracking and improvements in stem design and biomaterials enhancing osseointegration, were substantial contributions to modern design rotating hinge (RH) knee prostheses. Such remarkable progress in implant design has significantly reduced force on the bone-implant interface and minimized prosthesis failure (Draganich et al., 1999; Deehan et al., 2008; Giurea et al., 2014; Farid et al., 2015; Neuhaus and Maier, 2022).

Indications for RH TKA are discussed controversially in recent literature. Several authors only supported its use in joint salvage procedures, owing to high complication rates and low survival (Guenoun et al., 2009). However, other studies reported encouraging mid-term results and recommendations to expand indications responding to the progressive evolution in implant designs (Petrou et al., 2004; Hossain et al., 2010). Long-term observations will be required to improve the knowledge on indications regarding RH TKA.

This study aims to investigate the clinical, functional, and radiological outcomes of a novel RH knee prosthesis EnduRo^®^ (B. Braun Aesculap AG, Tuttlingen, Germany) in complex primary and revision procedures throughout a minimum of 7 years. Short- and mid-term studies already showed promising results (Giurea et al., 2014; Böhler et al., 2017).

Therefore, we intended to evaluate the long-term results of this RH TKA system using a new kind of bearing material, a carbon-fiber reinforced poly-ether-ether-ketone (CFR-PEEK), which is the first to be introduced in a TKA implant. We assessed complication frequencies, complication types, incidences of complications and clinical outcomes after RH TKA.

## Methods

### Patients

Approval by the local ethics committee was obtained for this study (protocol number: 703/2009). Seventy-three patients, who received the EnduRo^®^ prosthesis between January 2008 and December 2013, were assessed prospectively with a minimum follow-up of 7 years. All patients gave informed consent before being included in this study. Sixteen patients died from causes unrelated to the surgical procedure, such as cardiovascular diseases, pulmonary diseases, or oncological diseases, and one patient was lost to follow-up during the observational follow-up period. This resulted in 56 consecutive patients eligible for further analysis with a minimum follow-up of 7 years, eligible for further analysis. The mean follow-up time was 8.9 years (range 7.1–11.9 years).

In 21 patients (37.5%), the implantation of the EnduRo^®^ prosthesis was performed as a primary procedure, due to osteoarthritis of the knee with severe varus or valgus deformity of more than 20° degrees, multidirectional instability, insufficiency of collateral ligaments or significant bone loss. Thirty-five patients (62.5%) received the EnduRo^®^ RH during revision surgery as a result of instability, aseptic loosening or periprosthetic joint infection (PJI). Differentiation between aseptic or septic loosening was specified by clinical and radiological examinations and the institution’s comprehensive diagnostic algorithm for PJI. This involves sterile puncture of the joint followed by white blood cell (WBC) count of the synovial fluid, broad-range polymerase chain reaction (PCR) and microbiological analysis, and further laboratory bloodwork with WBC, CRP, Il-6 and Fibrinogen.

Twenty revision cases (57.1%) were treated with RH TKA as a second stage procedure because of a PJI during the two-staged revision surgery. The other 15 patients (42.9%) underwent rTKA in a single stage procedure due to aseptic loosening with substantial bone loss and severe instability after primary TKA, including flexion and extension gap mismatch.

Patient’s comorbidities were summarized and scored applying the Charlson Comorbidity Index (CCI) (Charlson et al., 1987). Table 1 offers an overview on demographic data and indications of the patients.

**TABLE 1:** Patient demographic data stratified by primary and rTKA.

	Total	Primary	Revision	*p* value
n (%)	56	21 (37.5%)	35 (62.5%)	n.s
Female n (%)	40 (71.4%)	19 (90.5%)	21 (60.0%)	
Age (SD)	71.1 years (9.8)	70.6 years (11.5)	71.3 years (8.6)	
BMI (SD)	31.0 (7.3)	29.9 (5.5)	31.7 (8.2)	n.s
CCI (SD)	4.13 (1.7)	4.1	4.2	n.s
Duration of surgery (SD)	180.3 min (34.7)	165.7 min (20.8)	190.1 min (34.2)	*p* = 0.01
Indications for RH TKA				
Primary (Indications)	—	21 (37.5%) (varus valgus >20°, instability, bone defect)
Revision (Indications)			20 (57.1%) (PJI)
			15 (42.9%) (aseptic loosening)

### Implant Design and Surgical Technique

The EnduRo^®^ RH knee prosthesis with a novel bearing material was used in all cases. Biomechanical properties and implant design were described in previous publications (Giurea et al., 2014). Briefly summarized, novel features of the implant include a transmission of force from the femoral to the tibial component running through a high congruent polyethylene (PE) insert without weight-bearing of axes and bushings. The hinge mechanism contributes insignificantly to the weight-bearing, rather than stabilizing the frontal and sagittal forces.

The axis is embedded in bushings and flanges, which are out of PEEK Optima LT1 (Invibio, Thornton-Cleveleys, UK) with carbon-fiber reinforcement containing 30% polyacrylonitrile- (PAN-) based carbon (CFR-PEEK LT1 CA 30) (further referred to as CFR-PEEK), a biomaterial introduced in knee arthroplasty for the first time by this system. Bearing and flanges primarily do notcontribute to weight bearing, but provide stability to the joint in case of severe coronal or sagittal instability. CFR-PEEK in these components aims to reduce wear on these highly loaded components. Biomechanics of the knee can be restored through the design of the prosthesis, which enables 3° of hyperextension, 140° of flexion as well as ±12° of rotation. Cemented fixation of the epiphyseal component is mandatory; stems for femur and tibia are modular in length and offset and are available as cemented and cementless options. In cases with uncontained bone defects, femoral and tibial wedges are available to restore the joint line. A nickel and chromium-free alloy version of the TKA system was used if hypersensitivity was present.

All surgical procedures were performed through a midline skin incision and medial parapatellar arthrotomy with lateral patellar luxation. Intramedullary femoral and tibial alignment guides with resection blocks were applied to accurately execute osteotomies accurately. Flexion and extension gaps were balanced using dynamic tension spreaders. If the joint’s stability, tested with trail components, proved satisfactory, a tourniquet was activated for implantation. In all cases, a hybrid technique with cemented epiphyseal and metaphyseal fixation and cement-free stem fixation was applied. Pulsatile lavage of the bone was performed before cementation in vacuum technique with gentamicin-loaded PMMA (Palacos R + G, Heraeus, Hanau, Germany). The patella was routinely resurfaced, a lateral release was performed if necessary to improve patellar tracking if necessary. Before wound closure, the tourniquet was released and one to two intra-articular drains were placed and left postoperatively until day two. Mobilization under full weight-bearing with the support of two crutches started postoperatively from day one onwards under physiotherapeutic guidance. Crutches were kept for 6 weeks after surgery.

Patients received perioperative antibiotic prophylaxis with cefazolin or clindamycin, in case of verified penicillin allergy. In cases of two-stage revision due to PJI, the antibiotic therapy was adjusted based on prior microbiological analyses and detected pathogenic bacteria. Antibiotic therapy for two-stage revision was sustained for 6 weeks after second stage TKA. Thromboembolic prophylaxis was given throughout 6 weeks.

### Follow-Up and Clinical Examinations

Follow-up examinations took place during week 3 and 6, again after 3 months and 1 year, and repeated annually after that. During these visits, clinical and radiographic examinations were accomplished. Besides the clinical examination for ROM, further assessments included stability and pain, the Knee Society Score (KSS), the Western Ontario and McMaster Osteoarthritis Index (WOMAC), and the Oxford Knee Score (OKS) were performed. Two independent observers analyzed standard anteroposterior, lateral, axial and full leg radiographs. Radiographs were screened for signs of loosening including radiolucent lines, osteolysis, PE wear or implant migration.

Complications leading to revision surgery were categorized based on a previously described classification system (Giurea et al., 2014): Type 1, PJI; Type 2, periprosthetic complications (rupture of the extensor mechanism, periprosthetic fracture, patella luxation and wound healing disturbances; and Type 3, implant failure (aseptic loosening, PE wear, instability, breakage of bushings, axis or stems). This particular classification system is used to differentiate between failures associated with the prosthesis and the applied materials (Type 3) and failures with minor relation to material and prosthesis type (Type 1 or 2).

### Statistics

Descriptive statistics were used to display demographic data. Data were evaluated for distribution by the Kolmogorov-Smirnov method. Pre- and postoperative results were compared by paired sample *t*-test. Implant survival was evaluated for each type of complication (Type 1–3). Estimates of survival were performed by using models for competing risk analysis as proposed by Fine and Gray, where death was modeled as a competing event (Fine and Gray, 1999; Scrucca et al., 2007). Based on competing risks data, cumulative incidence functions with 95% confidence interval (CI) were calculated to for data visualization. Gray’s test was used to detect differences in survival between the primary and revision group and assess the effects of periprosthetic infection on the implant survival and to examine the influence of potential other risk factors for revision. *p*-values of less than 0.05 identified statistical significance. Statistical analyses were performed using R v4.1.0 (R Software), SPSS v26 (IBM Corporation) and GraphPad Prism 9 (Graphpad Software).

## Results

### Functional Outcome

**FIGURE 1:**
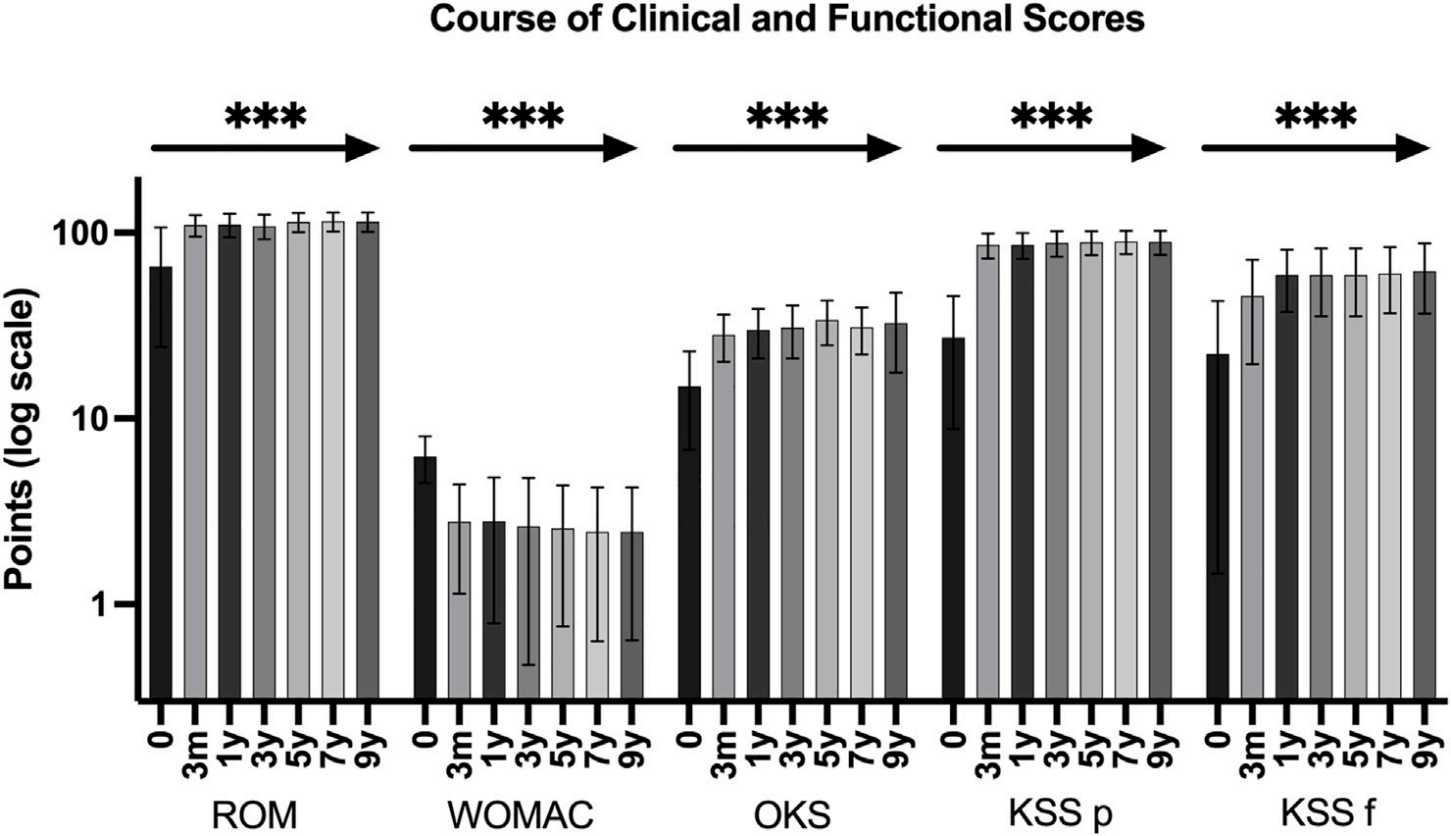
Clinical function and scores preoperative (0 months) and at 3 months, 1, 3, 5, 7, and 9 years after surgery. Clinical function and scores significantly improved from preoperative values to 3 months after surgery and sustained at the same level over the entire observational period. The asterisk and the arrow indicate the comparison between preoperative and each postoperative values separately (*** … *p* < 0.0001).

Clinical and functional parameters significantly improved after surgery and remained at that level throughout the observational period. The KSS improved from 27.3 (SD 18.5) preoperatively to 89.7 (SD 13.0) postoperatively (*p* < 0.0001) and the KSS for function improved from 22.3 (SD 20.79) to 60.3 (SD 23.3) (*p* < 0.0001), respectively. The WOMAC score improved from 6.26 (SD 1.77) before surgery to 2.45 (SD 1.82) after surgery (*p* < 0.0001) and the OKS improved from 14.9 (SD 8.15) to 30.9 (SD 8.75) (*p* < 0.0001). Further we found a significant improvement in active ROM from 65.0° (SD 41.4) to 115.1° (SD 13.4°) (*p* < 0.0001). Table 2; Figure 1 display an overview of clinical and functional scores. Clinical and functional scores significantly improved early after surgery, remaining at that level over the entire observational period without significant changes (Table 2; Figure 1).

**TABLE 2:** Detailed overview on clinical and functional scores from preoperative to 9 years after surgery. Results improved in the first 3 months after surgery and showed significant differences compared to preoperative values (*p* < 0.0001). Values stayed at the same level and did not significantly change thereafter (SD … standard deviation).

	Preop	3 months	1 year	3 years	5 years	7 years	9 years
ROM (SD)	65.0 (41.4)	109.9 (14.4)	110.6 (15.9)	108.5 (16.2)	114.2 (13.5)	115.1 (13.4)	114.7 (13.8)
Primary	92.8 (20.2)	110.3 (11.9)	112.6 (15.4)	112.6 (12.9)	115.0 (11.8)	115.6 (11.8)	114.6 (12.1)
Revision	47.7 (42.0)	109.7 (15.9)	109.3 (16.1)	105.0 (18.2)	113.5 (11.8)	114.8 (15.6)	114.7 (15.2)
WOMAC (SD)	6.26 (1.77)	2.78 (1.64)	2.80 (2.01)	2.63 (2.16)	2.56 (1.80)	2.45 (1.82)	2.45 (1.81)
Primary	6.46 (1.58)	2.70 (1.75)	2.65 (2.02)	2.06 (1.99)	2.29 (1.90)	2.18 (1.99)	1.92 (1.91)
Revision	6.14 (1.89)	2.83 (1.60)	2.90 (1.99)	3.09 (2.24)	2.81 (1.70)	2.69 (1.65)	2.81 (1.70)
OKS (SD)	14.92 (8.15)	28.19 (8.06)	29.96 (8.96)	30.79 (9.75)	31.41 (9.15)	30.94 (8.75)	32.66 (15.01)
Primary	14.15 (7.95)	29.00 (8.47)	31.53 (10.3)	34.58 (9.57)	33.94 (8.74)	33.33 (8.88)	33.00 (9.93)
Revision	15.41 (8.35)	27.69 (8.02)	28.89 (8.27)	27.65 (8.92)	29.00 (9.10)	28.80 (8.27)	32.41 (18.06)
KSS p (SD)	27.26 (18.50)	85.93 (13.39)	85.93 (13.64)	88.05 (13.75)	88.92 (13.20)	89.68 (13.01)	89.35 (13.09)
Primary	15.35 (15.35)	87.89 (12.23)	89.63 (10.80)	93.11 (8.51)	93.84 (8.62)	94.28 (8.49)	93.42 (10.19)
Revision	34.72 (17.17)	84.72 (14.60)	83.43 (14.94)	83.68 (15.98)	83.25 (15.2)	85.55 (15.07)	86.47 (14.40)
KSS f (SD)	22.25 (20.79)	45.63 (26.03)	59.27 (21.83)	59.10 (23.50)	59.10 (23.50)	60.26 (23.33)	62.24 (25.55)
Primary	21.75 (21.65)	45.28 (24.22)	55.79 (20.02)	62.63 (27.50)	61.84 (25.51)	61.67 (26.51)	62.92 (29.35)
Revision	20.94 (20.57)	48.86 (26.73)	61.96 (23.03)	56.36 (22.95)	56.50 (21.84)	59.00 (20.69)	61.76 (23.45)

### Cumulative Incidences and Competing Risk Analysis

**FIGURE 2:**
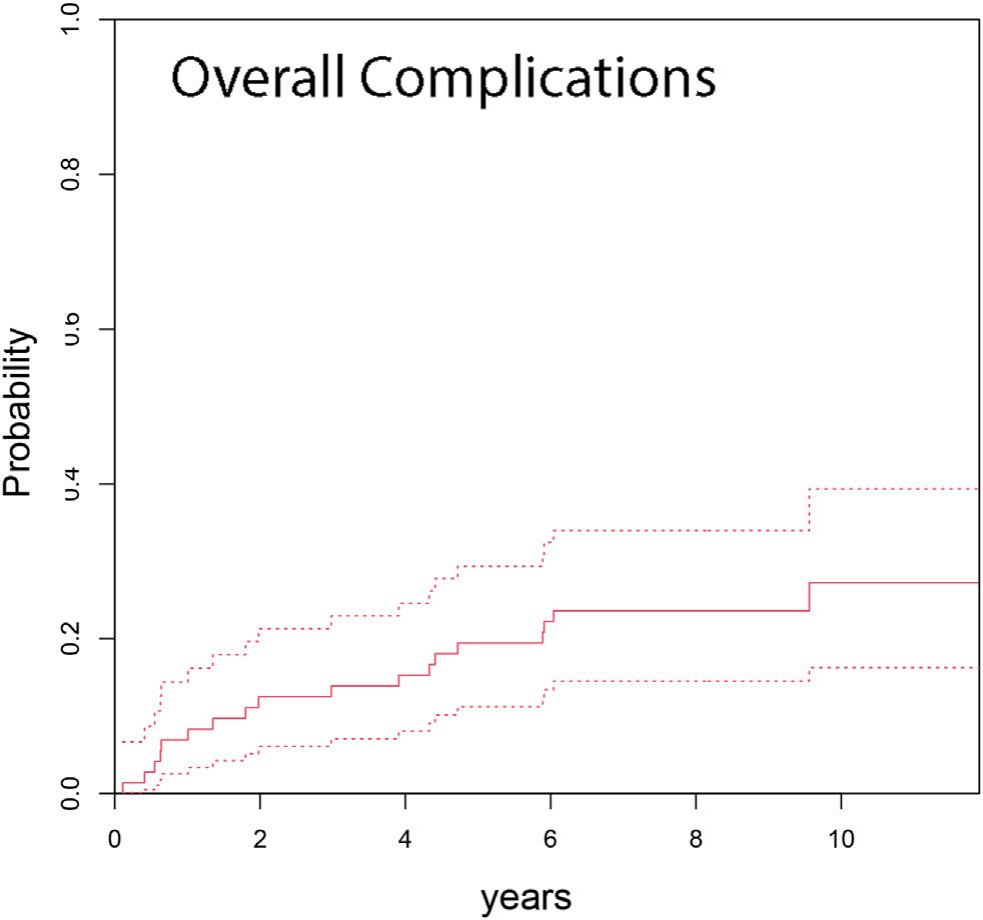
Cumulative incidence from competing risk data for any type of complication was 13.9% at 3 years, 19.4% at 5 years, and 23.6% at 7 years after RH TKA (dashed lines indicate 95% CI).

**FIGURE 3:**
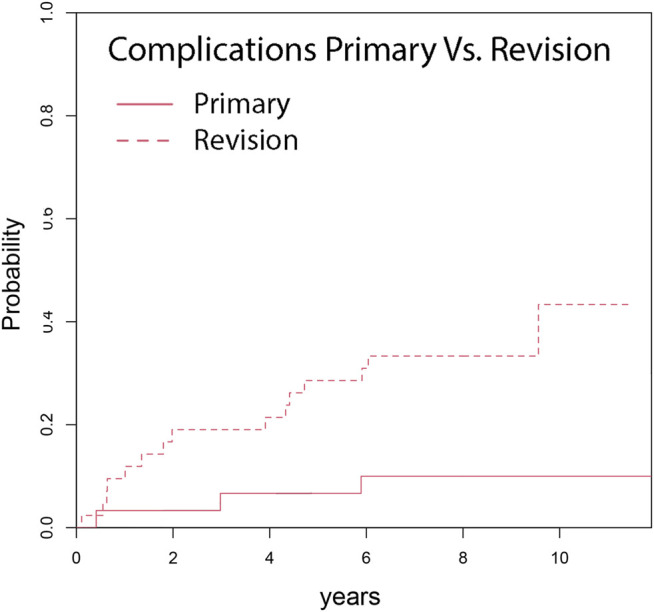
Cumulative incidence shows a significant differences in occurrence of complications of primary (10.0%) compared to rTKA (33.3%), (*p* = 0.011), after minimum 7 years follow up.

**FIGURE 4:**
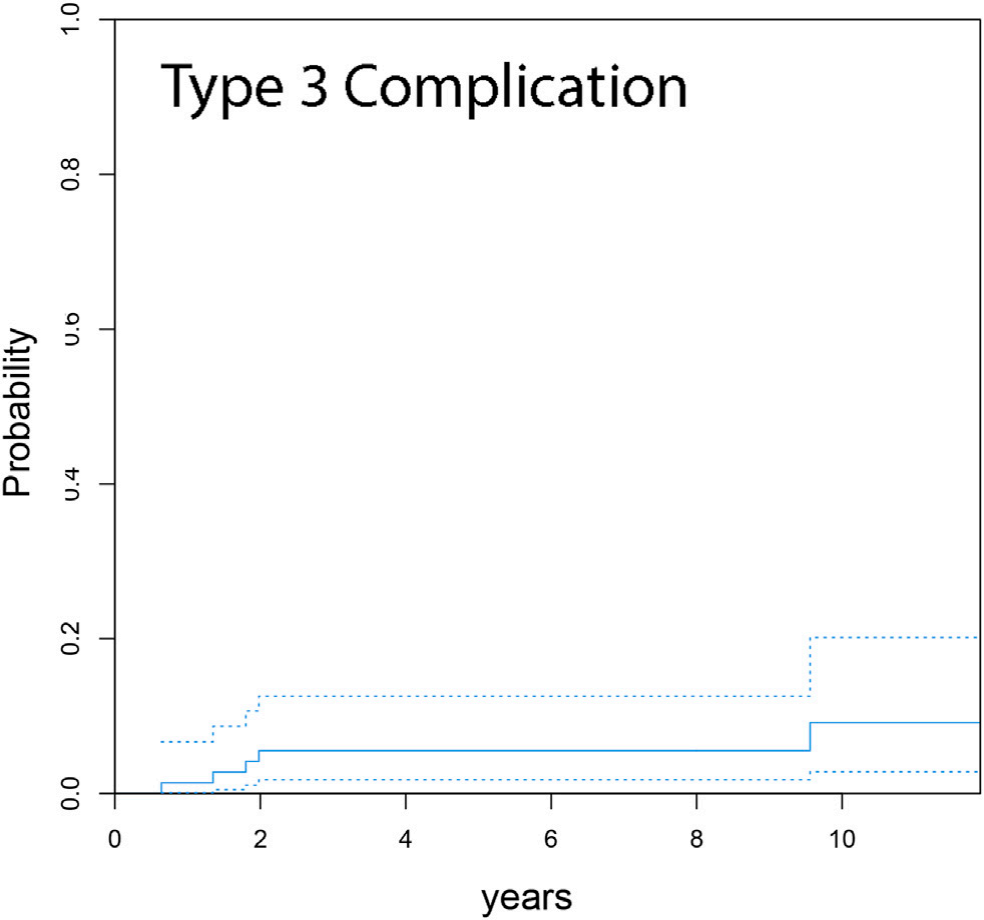
Cumulative incidence function from competing risk data for complications associated with implant failure (Type 3 complications) shows an occurrence of 5.6% at minimum 7 years follow up (dashed lines indicate 95% CI).

Competing risk analysis estimates an overall cumulative incidence for complications for any reason leading to revision surgery of 6.9% at 1 year, 13.9% at 3 years, 19.4% at 5 years and 23.6% at 7 and 10 years, respectively (Figure 2). The overall cumulative incidence in the primary group was 6.7% at 5 years and 10.0% at 7 years, respectively. The revision group’s cumulative incidence for complication was 9.5% at 1 year, 19.0% at 3 years, 28.5% at 5 years, and 33.3% at 7 years (Figure 3). Complications occurred significantly more frequently in the revision group compared to the primary group (*p* = 0.002). The cumulative incidence of implant-related complications (Type 3 complications) was 5.6% at 7 years (Figure 4).

### Complications and Risk Factors

**FIGURE 5:**
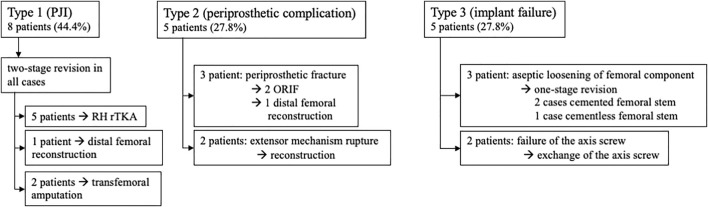
Complications leading to revision surgery after RH TKA occurred in 18 patients. The flow chart shows a comprehensive overview on the types of complication and the following procedure. Percentages in brackets relate to the number of all complications.

Complications leading to revision surgery occurred in 18 patients. Table 3; Figure 5 display an overview of types of complications. There was no complication recorded linked to a failure of the bearing material.

**TABLE 3:** Complications that occurred after RH TKA in primary and revision cases.

	Total (*n* = 55)	Primary (*n* = 20)	Revision (*n* = 35)
Complications overall n (%)	18 (100%)	3 (16.7%)	15 (83.3%)	*p* = 0.018
Type I n (%)	8 (44.4%)	1 (5.6%)	7 (38.9%)	
Type II n (%)	5 (27.8%)	2 (11.1%)	3 (16.7%)	
Type III n (%)	5 (27.8%)	0	5 (27.8%)	

Gray’s test revealed that rTKA significantly increased the risk for consecutive complication and revision surgery (*p* = 0.011). However, body mass index (BMI), CCI, duration of surgery sex and age had no significant influence on the development of any complication assessed by Gray’s test.

PJI occurred in eight patients. Six of these patients had a re-infection, after being previously treated by two-stage revision surgery. This results in a re-infection rate of 30%, considering that 20 patients were treated for PJI with two-stage revision surgery, as displayed in Table 1. Gray’s test revealed prior PJI as a highly significant risk factor for re-infection (*p* = 0.001).

## Discussion

Increasing frequencies of complex rTKA with substantial bone loss and severe ligamentous insufficiency limit the possibilities of sufficient and functional joint reconstruction. Consequently, RH TKA is a reliable option to restore joint function in these cases. In the current single-center study we present the results of 56 patients with a third-generation RH TKA, using a new bearing material for the first time in TKA. We found an overall complication-free survival of 76.4%. In total, 18 patients faced a complication that ultimately required revision surgery, resulting in a cumulative incidence of 23.6% at 7 years These findings are comparable with other observational studies that describe 10–40% complication frequencies and revision-free survival rates of 50–70%, making contemporary RH TKA a viable option in rTKA (Böhler et al., 2017; Cottino et al., 2017; Boelch et al., 2018; Kearns et al., 2018).

We used competing risk analysis to evaluate the risk of complications, as this might reflect a more realistic description of endoprosthetic survival in an elderly and multimorbid patient population (Fine and Gray, 1999; Gooley et al., 1999; Lacny et al., 2015).

The complication rate was significantly higher in patients with revision surgery than in the primary group. Previous PJI was the most influential risk factor for complications. These results confirm findings in current literature and underline that PJI remains one of the major challenges in revision arthroplasty (Pour et al., 2007; Hossain et al., 2010; Smith et al., 2013; Cottino et al., 2017; Staats et al., 2018; Röhner et al., 2019).

Gender, BMI, comorbidities and duration of surgery had no impact on the complication rate in our study. This might be due to our relatively small sample size (Bozic et al., 2014; Cherian et al., 2015).

The literature provides contradictory findings concerning the use of RH prostheses in complex primary TKA. Martin et al. described a long-term follow-up on 246 RH prostheses with a two times higher revision risk than conventional posteriorly stabilized or condylar constrained implants (Martin et al., 2016). Further, Badawy et al. showed an increased risk for revision for patients with RH TKA, compared to condylar constrained (CC) TKA. These findings are based on the Norwegian arthroplasty registry based on data starting from 1994 (Badawy et al., 2019). On the contrary Hossain et al. showed an overall survivorship for revision of 92.5% for RH TKA at 10 years with a superior satisfaction compared to CC prostheses (Hossain et al., 2010). In our study, the cumulative incidence for revision in complex primary TKA reaches rates of 10.0%, which translates to a revision-free survival of 90.0% at 7 years. We believe that registry data, which mainly comprises early RH designs, might not represent the outcome of contemporary RH TKA designs. In varus-valgus instability, CC TKAs can provide sufficient stability; however if additional anteroposterior instability is present, the use of constrained prostheses is inevitable (Kearns et al., 2018). When comparing both systems, these two different indications and biomechanical requirements for RH and CC prostheses should be considered. Therefore, long-term registry studies of modern RH TKA could clarify their impact on survival.

Implant-associated complications occurred in five patients, leading to a cumulative incidence of 5.6% at the end of the observational period. Further, it has to be pointed out that implant-related complications only occurred in the revision group, leading to a cumulative incidence of 9.5%. This could potentially be attributed to inferior bone quality and inferior soft-tissue balance compared to the primary group. However, a reliable hinge mechanism is crucial in RH TKA, since mechanical stress is high on this particular component. Failure of the axial screw was detected in two patients, representing 11.1% of all complications. Both failures occurred before the re-engineering of the axial screw system in the presented RH TKA system. A change to the new axial screw system was performed in both cases. No failure of the re-engineered axial screw mechanism has been observed since then. Wignadasan et al. found a comparatively low rate of 13% compliactions due to failure of the hinge, which matches our data before re-engineering the axial screw (Wignadasan et al., 2021).

Aseptic loosening occurred in three (16.7%) of the 18 complications, which corresponds to findings in current literature (Kearns et al., 2018; Wignadasan et al., 2021). This, on one side, may be attributed to the general improvement of fixation and cementation techniques in modern rTKA. The current literature provides evidence that the hybrid cementation technique provides a reasonably low risk for aseptic loosening; therefore, this approach was used in this study (Beckmann et al., 2011; Gómez-Vallejo et al., 2018). On the other hand, the low rate of loosening may be attributed to the implant design where transmission force travels from the femoral component to the tibial part via the polyethylene insert, whereas the axis is primarily not weight bearing and stabilizes the implant when higher frontal and sagittal forces occur. By that shear stress on the bone-implant interface can be remarkably reduced (Böhler et al., 2017).

Nevertheless, aseptic loosening only occurred in the revision group, showing that the risk for failure is more present in this setting than in the primary group.

The biomaterials requirements for bushing and flanges in RH prostheses is extremely high due to torsional moment and shear forces. Particle debris of worn bushing and flanges may contribute to adverse tissue reaction and consequently to periprosthetic loosening. Highly congruent CFR-PEEK was introduced as a biomaterial in RH TKA due to promising low rates of wear and debris in biotribologic studies (Grupp et al., 2013). First retrieval studies of CFR-PEEK bearing materials confirmed the anticipated wear. Especially some fragmented fibers and fiber/fiber fragment pull-outs in areas of wear with a reduced average roughness compared to the initial state were reported. However, the authors reported a higher variation of wear damage and changes in retrieved components than *in-vitro* tested material. Although the overall wear was not significantly different from *in-vitro* testing, the higher variance might result from more complex loading conditions during gait (Schierjott et al., 2016). Further studies report on the favorable effects of CFR-PEEK wear particles. Compared to ultra-high-molecular-weight polyethylene wear particles, CFR-PEEK wear particles were smaller than conventionally used materials. In cell culture experiments, CFR-PEEK particles showed no cytotoxic effect on fibroblast and macrophage lineage cells. These results suggest that, unlike other material debris, this material would not cause adverse tissue reactions such as tissue necrosis *in-vivo* (Howling et al., 2003; Scholes and Unsworth, 2009). Interestingly, wear patterns differed from conventionally used polyethylene bearings. CFR-PEEK particles showed no giant-cell reaction (Paulus et al., 2016). The clinical results in this study may confirm the promising preclinical data since failure linked to the CFR-PEEK bearing material could not be detected throughout this study. Additionally, there were no pathologic macroscopic or histologic findings of PEEK wear during revision surgery. However, since it is the first time CFR-PEEK was introduced in the RH prosthetic system used in this study, further clinical observation and monitoring are necessary.

Failure of the extensor mechanism represents a major concern in rTKA (Joshi and Navarro-Quilis, 2008). In our study population, extensor mechanism deficiency had an incidence of 3.6%. This rate might be lower than in previous reports, potentially caused by the novel design of the hinge mechanism. A periprosthetic fracture occurred in one case which corresponds with the literature (Gudnason et al., 2011).

It has been reported that using RH prostheses in complex TKA enables a significant increase in joint function and reduced pain (Springer et al., 2001; Pour et al., 2007; Theil et al., 2020). This study found a significant improvement in functional and clinical scores. Interestingly, patients showed a significant improvement of all measurements early at 3 months after RH TKA compared to preoperative values. Clinical and functional scores remained at a satisfactorily high level throughout the following observational period.

A limitations of our study is the sample size of 56 patients eligible for clinical follow-up because 18 patients died during the observation time of mean 8.9 years from causes unrelated to the RH surgery. This fact has to be considered when interpreting our results.

## Conclusion

The presented RH system provides reliable and durable reconstruction of the knee joint over a mean follow-up period of 8.9 years in cases of complex primary and rTKA. Competing risk analysis revealed a cumulative incidence for prosthesis failure of 5.6% after a mean observation time of 8.9 years. Long-term functional and clinical outcome improved early after surgery and remains highly satisfying over a long observational period.

## Data Availability

The raw data supporting the conclusion of this article will be made available by the authors, without undue reservation.
